# Probiotic supplement as a promising strategy in early tau pathology prevention: Focusing on GSK-3β?

**DOI:** 10.3389/fnins.2023.1159314

**Published:** 2023-03-22

**Authors:** Cassandra M. Flynn, Qi Yuan

**Affiliations:** Faculty of Medicine, Biomedical Sciences, Memorial University of Newfoundland, St. John’s, NL, Canada

**Keywords:** pretangle tau, neurofibrillary tangles, Alzheimer’s disease, probiotic, gut-brain axis, diabetes mellitus, GSK-3β

## Abstract

Neurofibrillary tangles (NFT) is one of the hallmarks of Alzheimer’s disease (AD). Recent research suggests that pretangle tau, the soluble precursor of NFT, is an initiator for AD pathogenesis, thus targeting pretangle tau pathology may be a promising early intervention focus. The bidirectional communications between the gut and the brain play a crucial role in health. The compromised gut-brain axis is involved in various neurodegenerative diseases including AD. However, most research on the relationship between gut microbiome and AD have focused on amyloid-β. In this mini review, we propose to target preclinical pretangle tau stages with gut microbiota interventions such as probiotic supplementation. We discuss the importance of targeting pretangle tau that starts decades before the onset of clinical symptoms, and potential intervention focusing on probiotic regulation of tau hyperphosphorylation. A particular focus is on GSK-3β, a protein kinase that is at the interface between tau phosphorylation, AD and diabetes mellitus.

## 1. Introduction

More than 55 million people worldwide are living with dementia, the number will nearly triple by 2050 (World Health Organization). Alzheimer’s disease (AD), the most common type of dementia, is characterized by two hallmarks: amyloid β (Aβ) plaques and neurofibrillary tangles (NFT) (Scheltens et al., 2016). To date, therapeutic approaches focusing on clearing Aβ has largely failed (Holmes et al., 2008; Tayeb et al., 2013; Mullane and Williams, 2020). Therapeutics that are successful in removing Aβ plaques have failed in improving cognitive function (Holmes et al., 2008; Tayeb et al., 2013). Antibody therapies focusing on soluble oligomers of Aβ, such as Aducanumab and Lecanemab, appear to have shown more promising effects in clinical trials (Ferrero et al., 2016; Panza et al., 2019; Shi et al., 2022). However, Aβ are often prevalent in aged brains without AD (Delaere et al., 1993). Targeting tau pathology seems to be a more promising approach, as tau pathology is highly correlated with cognitive dysfunction in AD patients (Spires-Jones and Hyman, 2014; Mullane and Williams, 2020). In this mini review, we discuss the importance of targeting pretangle soluble tau that starts decades before the onset of clinical symptoms, the link between tau hyperphosphorylation and aberrant GSK-3β activation, and potential prevention focusing on probiotic regulation of tau hyperphosphorylation *via* GSK-3β.

## 2. Why targeting pretangle tau

Seminal studies by Braak and Braak (1991) and Braak et al. (2011) have described the stereotypical patterns of tau pathology and progression in AD. These patterns were developed into a neurofibrillary tangle (NFT) staging (I-VI) system, and more recently, a pretangle tau staging system (a, b, c, Ia, and Ib) (Braak et al., 2011). Abnormally phosphorylated pretangle tau originates in the brain stem locus coeruleus (LC), spreads to other neuromodulatory nuclei before affecting the transentorhinal cortex. NFT is first formed in the entorhinal cortex (NFT stage I) and spreads to the limbic system including the hippocampus (stage II), affecting associative sensory cortices (stages III/IV), and eventually primary and secondary sensory cortices (stages V/VI) (Braak et al., 2011; Braak and Del Tredici, 2015). Abnormally phosphorylated pretangle tau appears to be the earliest sign of AD, preceding NFT (Braak et al., 2011). Thus preventing tau hyperphosphorylation could be ground zero for AD therapeutic strategies.

Neurofibrillary tangles has been considered a culprit of AD pathology. Cross-sectional studies of AD brains demonstrated a correlation of tangle accumulation with neuronal loss and dementia (Braak and Braak, 1997; Congdon and Duff, 2008). However, recent research suggests that soluble pretangle tau, including oligomers, are more toxic (Brunden et al., 2008; Congdon and Duff, 2008; Spires-Jones and Hyman, 2014). Key evidence supports this notion. Using computation modeling, Morsch et al. (1999) reported that in the CA1, tangle-bearing neurons survived for decades, thus NFT may not be the cause of cell death. In both higher order association cortex (Gomez-Isla et al., 1997) and hippocampus (Kril et al., 2002), the amount of NFT is correlated with disease duration but does not explain the degree of neuronal loss. Kril et al. (2002) examined the appearance of extracellular ghost tangles as an index of neuronal death *post*-NFT formation. Although marked neuronal loss (∼60%) was identified in AD brains, NFTs only accounted for 2–17% of total cell loss. A large proportion of neuronal death may occur prior to the formation of NFT.

Findings from animal tau models support the idea that NFT does not cause cognitive decline or neuronal death, and in some cases, may even be neuroprotective (Brunden et al., 2008; d’Orange et al., 2018). Synaptic loss and dysfunction preceded tangles in a P301s tau model (Yoshiyama et al., 2007). Using a mouse line expressing a repressible human tau, Santacruz et al. (2005) reported that suppression of transgenic tau following NFT formation successfully reversed neuronal loss and memory deficiency, while NFT continued to accumulate. In another study, the induction of a human wild-type tau (hTauWT) in rat brain resulted in tau hyperphosphorylation and neurotoxicity without aggregation. Surprisingly, co-expression of the hTauWT with a pro-aggregation tau peptide led to the formation of NFT but preserved neuronal survival (d’Orange et al., 2018). The reduction of soluble tau and Aβ was sufficient to ameliorate cognitive and behavioral deficits found in 3×Tg-AD mice, despite of the presence of NFTs and amyloid plagues (Oddo et al., 2006).

Recently, our laboratory has developed a pretangle tau model in rats that recapitulates some of the key features of Braak’s pretangle stages and preclinical pathology (Ghosh et al., 2019; Omoluabi et al., 2022). We seeded human tau pseudophosphorylated at 14 sites mostly in proline-rich regions (hTauE14) in the rat LC. The LC neurons expressing hTauE14 exhibited somatodendritic expression of the human tau, and hTauE14 spread to other neuromodulatory nuclei in the brain stem and the entorhinal cortex (Ghosh et al., 2019; Omoluabi et al., 2022). In the absence of NFT, hTauE14 rats showed impairment in olfactory associative discrimination, similar to olfactory dysfunction in pre-clinical AD (Conti et al., 2013; Devanand et al., 2015). LC fiber degeneration and neuronal loss were also observed and correlated with the severity of behavior deficiency, paralleling human observations (Gulyas et al., 2010; Theofilas et al., 2017). However, hTauWT seeding without pseudophosphorylation in the rat LC in another study showed negligible effects of neuronal toxicity (Kelberman et al., 2022). Together, the degree of abnormal tau phosphorylation appears to be a decisive factor in tau pathology.

## 3. Probiotic therapy reducing tau hyperphosphorylation *via* the GSK-3β pathway

As pretangle tau appears to be a crucial initiator in AD pathogenesis, strategies focusing on reducing tau hyperphosphorylation could be critical. A key feature in tau hyperphosphorylation is glycogen synthase kinase-3 (GSK-3), a proline-rich serine/threonine kinase (Sayas and Avila, 2021). GSK-3 is physiologically present in two isoforms GSK-3α and GSK-3β. The field has largely focused on the role of GSK-3β in tau pathology. Excessively activated GSK-3β contributes to the abnormal phosphorylation of tau, leading to the destabilization of microtubules, as seen in AD pathogenesis (Morris et al., 2011; Sayas and Avila, 2021). In addition to this, GSK-3 is a downstream regulator of other tau kinases and phosphatases, such as cyclin-dependent kinase 5 and protein phosphatase 1 and 2A (Bennecib et al., 2000; Plattner et al., 2006).

GSK-3β expression is up-regulated in the hippocampus of AD patients (Pei et al., 1999; Blalock et al., 2004). The active GSK-3β is initially found in pretangle neurons in the entorhinal cortex and extends to other brain regions in the same spatial sequence as tau pathology (Pei et al., 1999). Overexpression of GSK-3β in mice results in tau hyperphosphorylation, prevents induction of LTP (Hooper et al., 2007) and impairs spatial learning (Hernandez et al., 2002). Normalizing GSK-3β restores normal phosphorylated tau levels, reduces neuronal loss and cognitive deficit (Hernandez et al., 2002). Interestingly, GSK-3β overexpression is associated with tau hyperphosphorylation but not tangles in the hippocampus (Hernandez et al., 2002). Lithium, an inhibitor of GSK-3β, effectively reduces tau hyperphosphorylation (Munoz-Montano et al., 1997).

The bidirectional connections between the gut microbiota and the brain, termed the Microbiota-Gut-Brain axis, is a growing topic of interest in the pathogenesis of neurodegenerative diseases such as AD. Gut dysbiosis resulting from alterations in the composition and decreased biodiversity of the microbiome are observed in AD patients (Vogt et al., 2017; Sochocka et al., 2019) and AD rodent models (Nimgampalle and Kuna, 2017; Lee et al., 2019; Li et al., 2020). Changes in gut microbiota makeup as seen in AD can lead to increased intestinal barrier permeability and systemic inflammation (Stadlbauer et al., 2020). Combined with increased blood-brain barrier permeability in AD, this results in a pathway from the gut to the brain for neuroinflammatory cytokines, lipopolysaccharides (LPS), and toxic amyloid proteins to pass through (Lin et al., 2018; Pellegrini et al., 2018; Kowalski and Mulak, 2019). LPS, a cell wall component from gram-negative bacteria, is found in higher levels in AD patients, resulting in elevated pro-inflammatory mediators and further compromised blood-brain barrier, exacerbating neuroinflammation (Kim et al., 2021).

Dietary treatments, such as probiotics, present a therapeutic potential to gut dysbiosis and can provide a shift toward a healthier gut microbiome makeup (Gibson and Roberfroid, 1995; Krumbeck et al., 2016). Probiotics are defined as live microorganisms which confer a health benefit on the host by the World Health Organization. They have been shown to improve homeostasis of the internal microbiota, and maintain human intestinal health (Sanders, 2011; Sanders et al., 2011, 2018). When the number of beneficial bacteria rise, they compete for receptor sites with harmful bacteria and create a balance between harmful and beneficial bacterial species, thus providing a shift toward gut eubiosis (Sanders, 2011).

**FIGURE 1:**
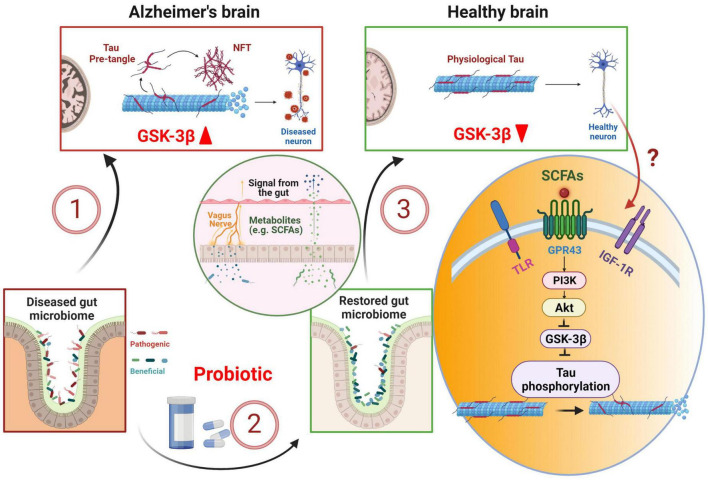
Probiotic therapy in early tau pathology prevention and treatment. Alzheimer’s disease is associated with dysbiosis in the gut, which in turn, can exacerbate tau pathology leading to tau hyperphosphorylation *via* GSK-3β pathway **(1)**. Probiotic supplement restores gut microbiome **(2)**, stimulates the release and transport of short-chain fatty acids (SCFAs) into the brain *via* enteric nerves and blood stream **(3)**. SCFAs stimulate PI3K/Akt pathway and down-regulates GSK-3β, thus preventing tau hyperphosphorylation. NFT, neurofibrillary tangle; TLR, Toll-like receptor; IGF-1R, insulin-like growth factor 1 receptor. Created with BioRender.com.

Probiotic therapy, which has been developed to reverse gut dysbiosis associated with AD (Vogt et al., 2017; Lin et al., 2018), has the potential of correcting tau hyperphosphorylation through GSK-3β suppression (Hooper et al., 2008; Lin R. et al., 2020; Figure 1). *L. plantarum* DP189 (Song et al., 2022) and *B. Breve* (Abdelhamid et al., 2022) strains of probiotics inhibits tau hyperphosphorylation in mouse models of AD. At mechanistic level, probiotic supplementation could exert its effect on GSK-3β and tau phosphorylation through PI3K/Akt signaling. Short-chain fatty acids (SCFAs) such as butyrate, produced by gut bacteria and subsequently released in the bloodstream, enhances gut barrier function and free fatty acid receptor FFA2/GPR43-mediated PI3K/Akt signaling in muscle cells (Tang et al., 2022). Probiotics or SCFAs can also act on PI3K/Akt signaling *via* other receptors such as insulin-like growth factor 1 receptor (IGF-1R) or Toll-like receptors (TLR) (Larraufie et al., 2017; Dang et al., 2018; Mohseni et al., 2021; Paveljsek et al., 2021). In the brain, *L. plantarum* gut administration results in an increase in Akt phosphorylation at S473, causing an elevated level of phosphorylated GSK-3β at S9 and subsequent inactivation of GSK-3β (Song et al., 2022). The inactivation of GSK-3β decreases tau phosphorylation at numerous proline-rich and non-proline sites (Hanger et al., 2009; Sayas and Avila, 2021). The precise route and mechanism of how gut probiotic supplement influences PI3K/Akt/GSK-3β signaling in the brain is not clear. However, *L. plantarum* has been shown to increase the abundance of butyrate-producing bacteria *Anaerotruncus* and *Faecalibacterium* (Wang et al., 2018). Therefore, it could mediate the brain effect through SCFAs circulating in the blood and binding to GPR43 receptors (Brown et al., 2003; Barki et al., 2022; Tang et al., 2022), TLR (Dang et al., 2018; Mohseni et al., 2021; Paveljsek et al., 2021), or IGF-1R *via* elevated serum IGF-1 (Endo et al., 2013; Yan et al., 2016; Mohseni et al., 2021). IGF-1R is widely expressed in the brain such as the hippocampus (Lin J. Y. et al., 2020). TLR is abundantly expressed in microglia, and to a lesser degree, neurons (Tang et al., 2007; Fiebich et al., 2018). GPR43 receptors are expressed in multiple tissues including neurons (Kimura et al., 2020; Barki et al., 2022). In another study, two strains of *L. Acidophilus* treatment in mice down-regulates GSK-3β gene expression (Yan et al., 2019). These studies suggest that probiotics can directly act on GSK-3β pathway and alleviate tau hyperphosphorylation. Furthermore, probiotic has been proven effective in treating gastric infection caused by *H. Pylori* (Aiba et al., 2015), which induces tau hyperphosphorylation in mouse hippocampal tissue (Wang et al., 2014; Uberti et al., 2022), *via* the GSK-3β pathway (Wang et al., 2014). Dysregulation of gut microbiota *via* gut-brain axis is associated with AD and probiotic supplement has the potential of correcting tau hyperphosphorylation through GSK-3β suppression. More extensive future research is in need to characterize the relationship between gut microbiota and tau hyperphosphorylation, especially in suitable animal models with GSK-3β induced tau hyperphosphorylation as a key feature.

## 4. The link between AD and diabetes *via* GSK-3β

Diabetes mellitus (DM), caused by lack of insulin, insulin resistance, or both, is considered a risk factor for AD (Zhang et al., 2018; Sun et al., 2020). AD has been referred to as “Type-3 diabetes” by researchers (de la Monte and Wands, 2008; Kroner, 2009) and the presence of DM nearly doubles an individual’s risk of developing AD (Leibson et al., 1997; Xu et al., 2004). Over 80% AD patients have type II DM or abnormal blood glucose level (Zhao and Townsend, 2009), suggesting a strong association between AD and DM.

Insulin has been recognized for its role in regulating Aβ protein and the generation of NFTs (Razay and Wilcock, 1994; Kroner, 2009). There is a feed-forward loop between insulin resistance and AD progression, resulting in higher levels of neuroinflammatory cytokines, reactive oxygen species, intracellular Ca^2+^, Aβ, GSK-3β activation, and tau hyperphosphorylation (Wei et al., 2021). It is also known that Aβ-facilitated tau phosphorylation by GSK-3 pathways can be mediated through the interference with insulin or wnt pathways (Townsend et al., 2007; Magdesian et al., 2008).

Dementia in the DM population shows significantly more tau accumulation than Aβ. A study by Hanyu and colleagues, showed 81% of patients with DM-related dementia showed an increase in tau protein, while only 39% showed Aβ accumulation through positron emission tomography imaging (Takenoshita et al., 2018). Impairment of insulin signaling is directly associated with tau phosphorylation. Hyperphosphorylated tau is found to be co-localized with increased insulin oligomers in both the hippocampus and the temporal cortex (Rodriguez-Rodriguez et al., 2017). The intraneuronal accumulation of insulin, increased insulin resistance and decreased levels of insulin receptors, are dependent on tau hyperphosphorylation and follow the progression of tau pathology (Rodriguez-Rodriguez et al., 2017). An siRNA mediated GSK-3β knockdown model showed a reduction of AD pathology through the restoration of the insulin signaling AMPK and Mapk3 pathways, resulting in improved cellular energy homeostasis, neuronal health, with reduced Aβ and tau formation in the cortex and hippocampus (Gupta et al., 2022).

Tau pathology, *via* GSK-3β over-activation, could be the specific link between diabetic patients and AD. Targeting the GSK-3β pathway through probiotics may provide a promising strategy to lower tau pathology and subsequently treat both AD and DM. In line with what has been shown in AD animal models, strengthening the gut-brain barrier through probiotic supplementation in a diabetes mouse model down-regulated GSK-3β levels compared to the diseased group without probiotic supplementation (Yan et al., 2019).

## 5. Conclusion and outlook

The findings reviewed here support the idea that soluble pretangle tau is a key player of tau pathology and highlight the need to target pretangle tau in AD prevention. Probiotic supplement could be a promising, natural, and non-invasive intervention to prevent pretangle tau formation. We highlight the roles of GSK-3β in mediating tau hyperphosphorylation and the effects of probiotic supplementation. We propose to further test probiotic treatments in pretangle tau models, as early intervention at preclinical stages may be a more feasible and fruitful approach for AD prevention.

Direct targeting of GSK-3β has its own limits. Concerns regarding GSK-3β as a ubiquitously expressed kinase, involved in several key cellular biological processes have been raised (Congdon and Sigurdsson, 2018). Two GSK-3β inhibitors AZD2558 and AZD1080 were brought to clinical trials, but were deemed not suitable for chronic AD treatment due to significant adverse side effects (Bhat et al., 2018). Tideglusib is the only GSK-3β inhibitor that has made to phase II clinical trials. Despite being associated with cognitive improvements and a reduction of cerebrospinal fluid levels of β-secretase in a subgroup of patients with mild AD, the clinical improvement was not significant (del Ser et al., 2013; Lovestone et al., 2015). While it remains challenging to bypass the widespread GSK-3β inhibition with pharmaceutical strategies, probiotic treatment has various additional beneficial effects (Vogt et al., 2017; Lin et al., 2018), thus providing a more holistic approach.

Future study could focus more on the sex difference of the GSK-3β signing. Sex differences in human AD (Podcasy and Epperson, 2016; Grimm and Eckert, 2017; Mosconi et al., 2017a,b; Laws et al., 2018; Yanguas-Casás et al., 2018) may also relate to hormone-mediated GSK-3β signaling. Perimenopause is the stage at which women show AD vulnerability (Brinton et al., 2015; Mosconi et al., 2017a; Neu et al., 2017; Pike, 2017). The neuroprotection role of 17β-estradiol has been linked to GSK-3β in animal models. A significant decrease in Aβ accumulation and hyperphosphorylated tau levels through the activation of 17β-estradiol has been associated with the inactivation of the GSK-3β pathway (Goodenough et al., 2005). It was also found that 17β-estradiol prevented GSK-3β induced neuronal apoptosis in hippocampal slice culture (Goodenough et al., 2005). This work adds additional support that estrogen can lower GSK-3β initiated tau phosphorylation. Keeping GSK-3β in check following menopause may be particularly important for women in AD prevention, and probiotic supplementation may provide some of the protections in this regard.

## Author contributions

CMF and QY contributed equally to the conception of the work and writing. Both authors contributed to the article and approved the submitted version.
